# 190. Incidence and Risk Factors for Clostridioides difficile disease (CDI) Progression from Polymerase Chain Reaction (PCR)+/Toxin- to PCR+/Toxin+ in Patients with Cancer: a Single Center Study

**DOI:** 10.1093/ofid/ofaf695.065

**Published:** 2026-01-11

**Authors:** Wonhee So, Maryam Jabri, Thu Phu, Justine Abella Ross, Jana Dickter, Rosemary She, Sanjeet S Dadwal

**Affiliations:** Western University of Health Sciences, duarte, CA; Western University of Health Sciences, duarte, CA; Western University of Health Sciences, duarte, CA; City of Hope National Medical Center, Duarte, California; City of Hope National Medical Center, Duarte, California; City of Hope, Duarte, California; City of Hope National Medical Center, Duarte, California

## Abstract

**Background:**

Two-step *Clostridioides difficile* infection (CDI) testing with PCR and EIA has improved diagnostic precision, but longitudinal significance of PCR+/toxin- (P+T-) test in immunocompromised patients is unclear. Our previous study showed P+/T- untreated patients had significantly higher P+/T+ CDI progression within 3 months compared to P+/T- patients who received CDI treatment based on signs or symptoms of CDI without alternate explanation. This study aimed to analyze the risk factors for progression over 3 months from P+/T- to a P+T+ test result.

**Figure:**
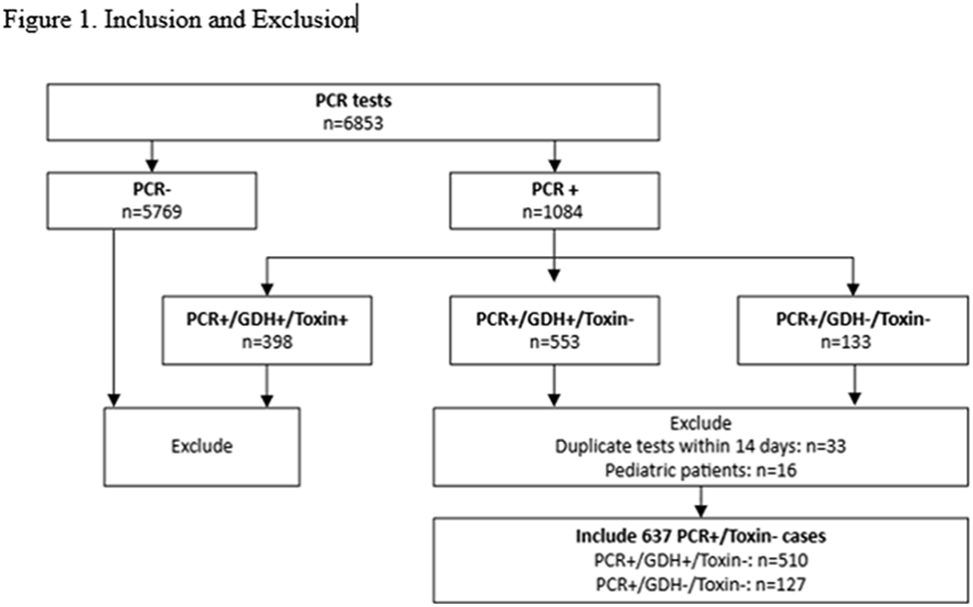

**Figure:**
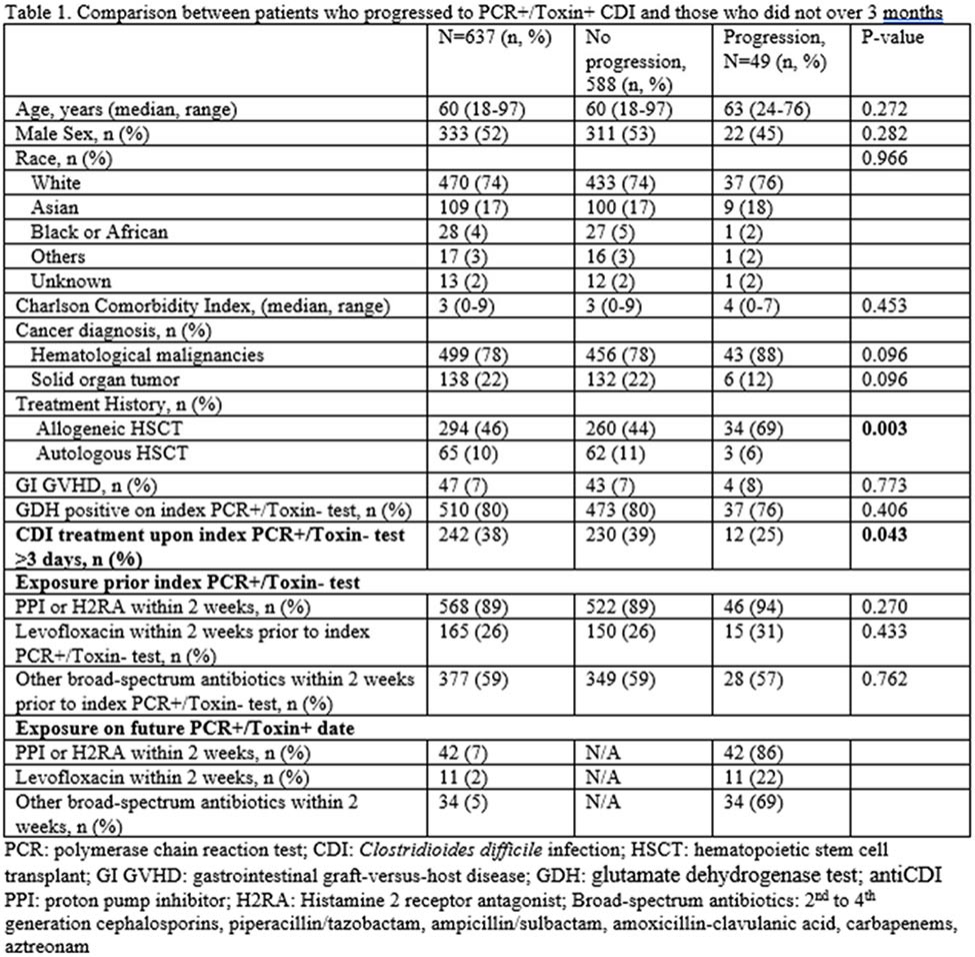

**Methods:**

Retrospective review of adult, hospitalized patients with P+T- from 8/18/2021-8/17/2024 was performed (Figure 1). Duplicate tests ≤14 days were excluded. Patient demographics, glutamate dehydrogenase (GDH) test positivity, CDI treatment defined as receiving ≥3 days of CDI treatment at the time of index P+T- test, exposure to proton pump inhibitors/histamine 2 receptor antagonist/ antibiotics were compared between P+T+ progression group and no progression group (Table 1). Logistic regression was performed to identify factors associated with P+T+ CDI progression using SPSS v. 29.

**Results:**

Among 637 P+T– cases with median age 60, male 52%, hematological malignancies 78%, 38% received CDI treatment and 8% (49/637) progressed to P+T+ CDI within 3 months (Figure 1, Table 1). Compared to those who did not progress, patients who developed confirmed P+T+ CDI within 3 months were more likely to have undergone allogeneic hematopoietic stem cell transplant (HSCT, 69% v. 44%, p=0.003) and less likely to have received CDI treatment at index P+T- result (25% v. 39%, p=0.043). Multivariable regression analysis controlled for age, GDH test positivity, and CDI treatment at the time of index P+T-, identified only allogeneic HSCT (OR 3.020, p< 0.001) as a predictor for P+T+ CDI progression.

**Conclusion:**

Increased risk of P+T+ CDI progression from P+T- among allogeneic HSCT patients highlights the importance of close monitoring and preventive strategies in high-risk populations. These findings suggest a potential role for prophylaxis as CDI in allogeneic HSCT is independently associated with increased non-relapse mortality and needs to be studied.

**Disclosures:**

All Authors: No reported disclosures

